# Retraction Note: PIK3R3, part of the regulatory domain of PI3K, is upregulated in sarcoma stem-like cells and promotes invasion, migration, and chemotherapy resistance

**DOI:** 10.1038/s41419-024-06609-6

**Published:** 2024-03-19

**Authors:** Changhwan Yoon, Jun Lu, Sandra W. Ryeom, M. Celeste Simon, Sam S. Yoon

**Affiliations:** 1 Department of Surgery, Memorial Sloan Kettering Cancer Center, New York, NY USA; 2 Department of Gastric Surgery, Fujian Medical University Union Hospital, Fujian, China; 3 Department of Cancer Biology, University of Pennsylvania, Philadelphia, PA 19104 USA; 4 Abramson Family Cancer Research Institute, Perelman School of Medicine, University of Pennsylvania, Philadelphia, PA 19104 USA

**Keywords:** Cancer stem cells, Oncogenes, Sarcoma

Retraction to: *Cell Death & Disease* 10.1038/s41419-021-04036-5, published online 29 July 2021

The Editors-in-Chief have retracted this article after concerns were raised about some of the data reported. Specifically:

- In Fig. 2a, the HT1080 spheroid images appear to have been published in a previous article with common authors [1], where they are described differently.
- In Fig. 2b, sh.PIK3R3 panels appear to be the same for both HT1080 Spheroids and SK-LMS-1 Spheroids.
- In Supplementary Fig. S2, the panel for invasion monolayer SK-LMS1 in S2a appears to be the same as the panel for invasion + HT1080 Spheroids in S2d. Further, the panel for Migration Spheroids DDLS8817 in S2a appears to have been previously published in a paper with common authors [2], in which it is described differently.
- In Supplementary Fig. S3b, the panel for HT1080 CD133+; LY294002 appears to have been previously published in a paper with common authors [3], where it is described differently.
- In Supplementary Fig. S4, HT1080 and SK-LMS-1 panels appear to have been previously published in a paper with common authors [4], where they are described differently.

Additionally, the authors were unable to provide documentary evidence that they received ethics approval for the animal work reported in this article. The Editors-in-Chief no longer have confidence in the reliability of the results and conclusions of this article.

M. Celeste Simon agrees with this retraction. Sam S. Yoon agrees with this retraction but disagrees with the retraction wording. The Publisher was unable to confirm current contact details for Changhwan Woon. Jun Lu and Sandra W. Ryeom did not respond to correspondence from the Publisher about this retraction.
